# All-Inside and Conventional Techniques in Anterior Cruciate Ligament Reconstruction: A Retrospective Comparison Study

**DOI:** 10.3390/jcm15041404

**Published:** 2026-02-11

**Authors:** Evren Karaali, Osman Çiloğlu, Bedirhan Sarı, Oğuzhan Çiçek, Özhan Pazarcı, Mesut Uluöz, Furkan Kanca

**Affiliations:** Department of Orthopedics and Traumatology, Adana City Training and Research Hospital, Adana 01230, Turkey; osmanciloglu@gmail.com (O.Ç.); bedirhan.sarimd@gmail.com (B.S.); oguzhan.cicek21@hotmail.com (O.Ç.); dr.pazarci@gmail.com (Ö.P.); mesutuluoz@hotmail.com (M.U.); furkanca0923@gmail.com (F.K.)

**Keywords:** anterior cruciate ligament reconstruction, all-inside technique

## Abstract

**Objective**: The aim of the study was to compare postoperative pain, functional recovery, knee stability and complication profiles between the all-inside technique and the conventional full tibial tunnel technique for anterior cruciate ligament (ACL) reconstruction. **Methods**: This retrospective comparative cohort study included 104 patients who underwent primary ACL reconstruction between 2018 and 2020. Surgical technique allocation was non-randomized. Patients were divided into two groups based on the surgical technique employed; the conventional full tibial tunnel group (*n* = 58) and all-inside group (*n* = 46). Hamstring tendon autografts were used in all cases, and the procedures were performed by the same surgical team to ensure consistency. Clinical outcomes were evaluated using the Visual Analog Scale (VAS) for pain, Lysholm knee score and International Knee Documentation Committee (IKDC) score. Knee stability was assessed with the Lachman and pivot-shift test. Additionally, postoperative complications and revision rates were recorded. **Results**: The study included 104 patients, with 58 treated using the conventional technique and 46 using the all-inside technique. Baseline characteristics, trauma mechanisms, and follow-up duration were comparable between groups (*p* > 0.05). Four patients in each group underwent revision surgery and excluded from the final analysis. Revision rates were comparable between groups (6.9% vs. 8.6%) and were considered descriptively when interpreting outcomes. The preoperative VAS, Lysholm and IKDC scores were comparable between groups. At 3 months, the all-inside group demonstrated significantly lower VAS pain scores and higher Lysholm and IKDC scores (with moderate-to-large effect sizes) (all *p* < 0.01). No significant differences were observed at the 12-month or final follow-up. Postoperative knee stability was comparable between groups, whereas anterior knee pain was significantly less frequent in the all-inside group (*p* < 0.001). Moreover, patients treated with the all-inside technique returned to sports significantly earlier than those treated with the conventional technique (13.25 ± 2.70 vs. 16.40 ± 5.85 months, *p* < 0.001; Cohen’s d = 0.66). However, the proportion of patients who returned to their preinjury sports level was comparable between the two groups (81.0% vs. 83.3%, *p* = 0.78). **Conclusions**: The all-inside ACL reconstruction technique was associated with superior early pain relief and short-term functional outcomes compared with the conventional technique, while achieving comparable knee stability and mid-term clinical results. These advantages occur without increasing complications or revision rates. These findings support the all-inside technique as patient-centered, optimizing early recovery without sacrificing mid-term clinical durability. These findings should be interpreted in the context of the non-randomized retrospective study design.

## 1. Introduction

Anterior cruciate ligament (ACL) injury is a common cause of knee instability, for which surgical reconstruction is the preferred treatment [1]. The all-inside reconstruction technique is increasingly popular owing to its minimally invasive nature and potential to improve early postoperative comfort [2]. Recent systematic reviews have demonstrated that this technique provides reliable functional and biomechanical outcomes [3].

Several studies have examined various surgical techniques for ACL reconstruction [4,5]. Various factors influence the choice of reconstruction method, including patient anatomy, injury mechanism, surgeon experience, and graft selection [6]. Conventional reconstruction techniques provide reliable and stable functional outcomes, as shown in prospective follow-up studies. However, these approaches may increase postoperative pain, bone loss, and the risk of fatigue-related complications due to full tibial tunnel drilling and extensive surgical exposure [7]. Recent advances in arthroscopic techniques have led to increased interest in less invasive approaches to ACL reconstruction. The all-inside technique is a tissue-preserving alternative that limits bone removal, reduces graft length requirements and maintains cortical integrity on both the femoral and tibial sides. This approach contributes to improved early postoperative comfort and functional recovery by minimizing surgical trauma associated with full tibial tunnel drilling. Nevertheless, despite its increasing clinical use, whether these early advantages result in durable and long-term clinical outcomes remains unclear [8,9]. Specifically, it remains unclear whether the early postoperative benefits attributed to the all-inside technique translate into durable midterm outcomes without compromising knee stability when compared with conventional full tibial tunnel reconstruction. The best timing of surgery, type of grafts to use, choice of a fixation device, and method of rehabilitation are debatable [8]. The present study focuses on a young and physically active population aged 18–28 years in order to reduce clinical heterogeneity related to age, activity level, and degenerative changes. While this approach enhances internal validity and allows more precise comparison of surgical techniques, it may limit the generalizability of the findings to older or less active patient populations. We hypothesized that the less-invasive all-inside ACL reconstruction would reduce early postoperative pain and accelerate functional recovery without compromising knee stability or midterm outcomes compared with the conventional technique. Knee stability was assessed using standardized clinical examination tests, which reflect functional stability in routine clinical practice, although instrumented laxity measurements were not available.

## 2. Materials and Methods

### 2.1. Study Design and Patient Selection

The local ethics committee approved this retrospective study. Informed consent was obtained from all participants or waived in accordance with the study’s retrospective nature. In total, 104 patients who underwent ACL reconstruction between 2018 and 2020 were initially included. Patients were divided into two groups based on the surgical technique used: conventional full tibial tunnel technique (*n* = 58) and the all-inside technique (*n* = 46). Selection of the surgical technique was primarily based on surgeon preference and evolving institutional practice patterns during the study period, rather than predefined patient-specific criteria. Inclusion criteria were a primary ACL rupture confirmed by clinical examination and magnetic resonance imaging (MRI), patients age 18–28 years, active sports participation and compliance with follow-up visits. A minimum follow-up period of 48 months was required. Patients meeting the minimum follow-up requirement were also evaluated at predefined interim time points (3 and 12 months), and missing data at these intervals were handled by analyzing available-case data. Patients presenting with multi-ligament knee injury, a history of knee surgery, advanced concomitant meniscal or chondral lesions were excluded. Advanced lesions were defined as high-grade chondral defects (Outerbridge grade III–IV) or complex meniscal pathology requiring extensive repair or meniscectomy that could independently influence postoperative clinical outcomes. During screening, a total of eighteen patients were excluded for these reasons, including four patients with advanced chondral defects and fourteen patients with meniscal lesions requiring extensive repair or meniscectomy. These findings were identified based on preoperative magnetic resonance imaging and/or intraoperative assessment. During follow-up, eight patients (four from each group) who underwent revision ACL reconstruction were excluded from the final analysis. Consequently, the final study population included 54 patients in the conventional group and 42 patients in the all-inside group. Comparative analyses were performed between the two groups. Data were obtained from patient medical records. Demographic and clinical variables were systematically recorded; age, sex, mechanism of injury, functional outcome scores, pain scores, clinical stability test results, and the presence of anterior knee pain.

### 2.2. Surgical Techniques

All procedures were performed by the same surgical team using standardized arthroscopic techniques and ACL reconstruction instruments and fixation systems (Smith & Nephew, Andover, MA, USA). Hamstring tendon autografts were harvested and prepared according to established protocols. As part of standard perioperative management, all patients received intravenous tranexamic acid (1 g) intraoperatively, followed by oral tranexamic acid (500 mg tb, 25 mg/kg) for three days postoperatively.

#### 2.2.1. Conventional Technique

**Figure 1:**
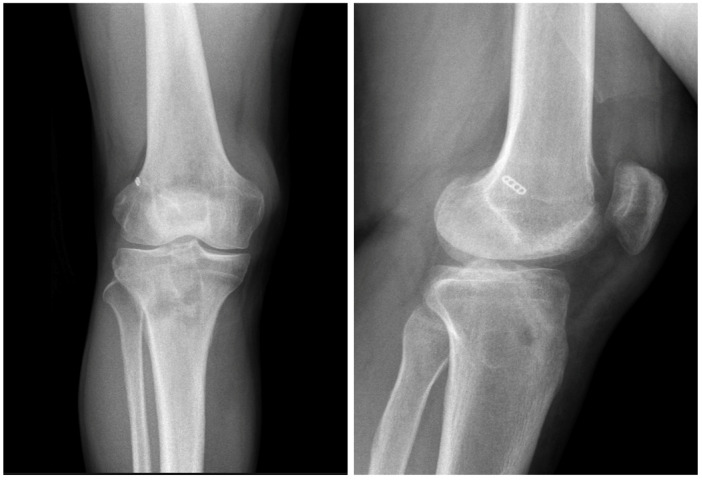
Postoperative AP knee radiographs after conventional ACL reconstruction.

In the conventional group, ACL reconstruction was conducted using a full tibial tunnel technique. A full-length tibial tunnel was created in an antegrade manner, followed by the creation of a femoral tunnel. Femoral fixation was achieved using a cortical suspensory device (EndoButton), while tibial fixation was secured with an interference screw (Figure 1). For this technique, a longer hamstring tendon autograft was required to span the full trans osseous tibial tunnel. In this group, both the semitendinosus and gracilis tendons were harvested to obtain sufficient graft length for conventional full-length tibial tunnel reconstruction. The graft was prepared in a standard multistranded configuration to ensure adequate length and diameter for conventional tunnel fixation. Conventional ACL reconstruction was performed using standard full-length tibial tunnel drilling, with femoral and tibial fixation achieved using interference screw–based fixation according to routine institutional practice.

#### 2.2.2. All-Inside Technique

**Figure 2:**
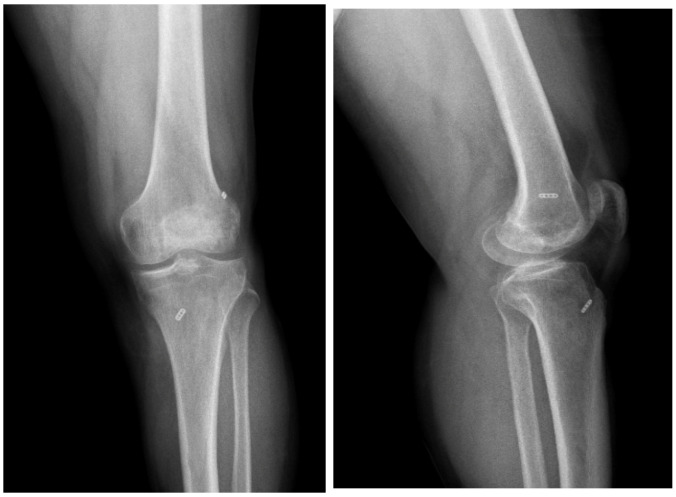
Postoperative AP knee radiographs after all-inside ACL reconstruction.

In the all-inside group, ACL reconstruction involved a socket-based technique with retrograde drilling. Femoral and tibial sockets were created using a retrograde drilling system, without creating a full tibial tunnel. Cortical bone was preserved on both sides, and graft fixation was achieved using cortical suspensory devices on the femoral and tibial sides (Figure 2). This technique utilized a shorter hamstring tendon autograft, prepared as a four-stranded (quadrupled) short graft in accordance with established all-inside reconstruction principles. This graft configuration reduces graft length requirements while maintaining adequate graft diameter and fixation strength. In the all-inside group, semitendinosus tendon autografts were prepared as four-stranded (quadrupled) grafts, with a target graft diameter of ≥8 mm in all cases to minimize the risk associated with thin graft constructs. The gracilis tendon was preserved in all patients undergoing the all-inside technique. Femoral and tibial fixation was achieved using cortical suspensory devices in all all-inside reconstructions, with retrograde drilling performed in a standardized manner.

### 2.3. Postoperative Rehabilitation

Patients followed a standardized postoperative rehabilitation program, with adjustments made for individual factors and postoperative recovery speed. No knee brace was used following surgery. Early rehabilitation focused on passive and active-assisted range-of-motion exercises, with emphasis on restoring full knee extension. Closed kinetic chain strengthening exercises began gradually during initial rehabilitation. Partial weight-bearing was permitted immediately after surgery and progressed to full weight-bearing based on patient tolerance. Proprioceptive and neuromuscular training were implemented during the intermediate phase and sport-specific and agility exercises commenced after 6 months. Return to sports was recommended after 9 months, provided they met predefined functional recovery criteria. Although a standardized rehabilitation framework was applied, progression through rehabilitation phases was individualized based on patient tolerance and functional recovery.

### 2.4. Outcome Measures

Patients were evaluated preoperatively, at predefined interim time points (3 and 12 months), and at final follow-up using the following outcome measures:

- Visual Analog Scale (VAS) pain score
- Lysholm knee score
- International Knee Documentation Committee (IKDC) score
- Presence of anterior knee pain (defined as patient-reported pain localized to the anterior aspect of the knee during daily activities or kneeling)
- Stability tests (Lachman and pivot-shift)
- Time to return to work
- Time to return to sport

All patient-reported outcome measures were administered using previously validated Turkish versions of the respective questionnaires. Time to return to sport was defined as the interval from surgery to unrestricted participation in preinjury sports activities. Objective return-to-sport readiness tools were not routinely available during the study period, and return was determined based on clinical and functional assessment.

Knee stability was assessed using standardized clinical examination tests. Lachman testing was performed during routine outpatient clinical follow-up visits and graded according to the degree of anterior tibial translation and the quality of the endpoint (negative, grade I, II, or III). Pivot-shift testing was assessed intraoperatively under anesthesia, which is considered to improve reliability, and was performed by the primary operating surgeon in all cases. Pivot-shift results were recorded as positive or negative based on the presence of a palpable subluxation or clunk during dynamic testing. To ensure consistency, all stability assessments were conducted by the same investigator. Objective instrumented laxity measurements, such as KT-1000 testing, were not routinely available during the study period.

### 2.5. Statistical Analysis

Statistical analyses were conducted using IBM SPSS Statistics version 26.0 (IBM Corp., Armonk, NY, USA). The normality of continuous variables was assessed using the Shapiro–Wilk test. Normally distributed data were reported as mean ± standard deviation and compared using the independent samples *t*-test, while nonnormal variables were analyzed using the Mann–Whitney U test. Categorical variables were summarized as frequencies and percentages and evaluated using the chi-square test or Fisher’s exact test. Clinical outcome measures, including VAS, Lysholm knee, and IKDC scores, were compared between groups preoperatively and postoperatively. Statistical significance was set at *p* < 0.05 (two-tailed).

## 3. Results

Initially, 104 patients were enrolled, including 58 patients treated with the conventional technique and 46 with the all-inside technique. The two groups were comparable in baseline demographic characteristics (e.g., age and sex distribution), trauma mechanisms, and follow-up duration, with no significant differences identified between groups (*p* > 0.05). Baseline comparisons were limited to available demographic and clinical variables recorded in the study database. Revision ACL reconstruction was required in four patients per group, with no significant difference between techniques. Revision rates were therefore analyzed as clinical outcomes rather than exclusion criteria and were comparable between techniques. When revision cases were evaluated using an intent-to-treat perspective, failure rates remained comparable between the conventional and all-inside techniques (6.9% vs. 8.6%). Review of revision indications revealed that the majority of failures were associated with traumatic reinjury during sports participation rather than technical errors, fixation failure, or graft-related complications. No systematic pattern suggesting technique-specific mechanical inadequacy was identified. These patients were excluded from the final functional outcome analysis. Revision surgery was the only complication systematically recorded and analyzed; no additional major complications requiring surgical intervention were observed during follow-up (Table 1).

Preoperative clinical outcome measures were comparable between the conventional and all-inside groups, with no significant differences observed in VAS, Lysholm knee, or IKDC scores (VAS: *p* = 0.33; Lysholm: *p* = 0.340; and IKDC: *p* = 0.36). At the 3-month follow-up, patients treated with the all-inside technique demonstrated significantly lower VAS scores and significantly higher Lysholm and IKDC scores compared with those treated with the conventional technique with moderate-to-large effect sizes favoring the all-inside technique (*p* < 0.01). At the 12-month and final (48-month) follow-up evaluations, no significant differences were found in VAS, Lysholm, or IKDC scores between the two groups (*p* > 0.05). Moreover, time to return to sport was shorter in the all-inside group (13.25 ± 2.70 months) than in the conventional group (16.40 ± 5.85 months) (*p* < 0.001; Cohen’s d = 0.66). However, the proportion of patients who returned to their preinjury level of sport was comparable between the all-inside and conventional groups (81.0% vs. 83.3%; *p* = 0.78) (Table 2).

Preoperative knee stability assessments showed no significant differences in pivot-shift positivity rates or Lachman test grade distributions between groups (*p* > 0.05) (Table 3A,B). At the 3-month postoperative evaluation, the pivot-shift positivity rates and Lachman test grade distributions remained comparable between the two groups (*p* > 0.05). In contrast, anterior knee pain was significantly less frequent in the all-inside group at the 3-month, 12-month, and final follow-up (*p* < 0.001 for all comparisons). In addition, the lower incidence of anterior knee pain in the all-inside group persisted throughout all follow-up periods, underscoring the consistency and clinical relevance of this finding. The magnitude of early pain reduction at 3 months was clinically meaningful, with a large effect size (Cohen’s d > 0.8) favoring the all-inside technique. Anterior knee pain was assessed consistently at each follow-up using patient-reported symptoms, which may partly explain the relatively high prevalence observed in the conventional group (Table 3A,B).

Between-group comparisons were primarily based on mean differences and effect sizes rather than adjusted estimates.

## 4. Discussion

This study compared postoperative pain, functional outcomes, knee stability, and complication rates between the all-inside technique and conventional full tibial tunnel technique for ACL reconstruction. The main finding of the study is that the all-inside technique offers notable advantages during the early postoperative period, particularly regarding pain relief and functional recovery, while demonstrating comparable knee stability and midterm clinical outcomes when compared with the conventional approach. Early advantages observed with the all-inside technique were primarily confined to the early postoperative period, whereas midterm clinical and functional outcomes converged between techniques over time. Prior studies focused on short-term outcomes in mixed patient populations; however, this study provides midterm follow-up data in a homogeneous cohort of young, physically active patients. Furthermore, consistently assessing anterior knee pain at multiple postoperative time points provides a patient-centered perspective that remains underrepresented in existing literature.

These findings indicate that reducing surgical invasiveness influences the early postoperative period rather than long-term clinical performance. The observed benefits may be attributed to bone stock preservation and reduced soft tissue disruption, facilitating faster recovery without compromising mechanical stability. In the current cohort, patients treated with the all-inside technique exhibited significantly lower postoperative pain scores during early follow-up. This pain reduction was most evident during early recovery and did not persist, reinforcing that the benefits of all-inside approach are predominantly short-term. Given that anterior knee pain frequently causes patient dissatisfaction and delayed return to sport, the sustained reduction observed with the all-inside technique represents a clinically relevant advantage beyond early pain control.

Functional outcomes showed a similar temporal pattern. Although preoperative functional scores were comparable between groups, the all-inside group revealed significantly higher Lysholm and IKDC scores at the 3-month follow-up, indicating faster early functional recovery. These findings are consistent with those of prior randomized and retrospective studies reporting improved short-term functional outcomes with all-inside or minimally invasive ACL reconstruction [2,4,10]. However, at the 12-month and final follow-up evaluations, functional scores were comparable between groups, indicating that both techniques provide equivalent midterm functional results. This observation aligns with large-scale meta-analyses and systematic reviews demonstrating that functional outcomes converge over time, regardless of surgical technique [1,6]. The earlier return to sport observed with the all-inside technique was not associated with a lower rate of return to preinjury sport level, demonstrating that accelerated recovery was not achieved at the expense of functional readiness. Earlier return to sport should not be interpreted as inherently superior in the absence of objective readiness assessments, and clinical judgment remains essential when determining safe timing for return to athletic activity.

The significantly shorter time to return to sport observed in the all-inside group may be partially explained by differences in surgical invasiveness between techniques. Conventional ACL reconstruction requires full-length tibial tunnel drilling, which results in greater bone and soft tissue trauma compared with socket-based all-inside reconstruction. This increased surgical exposure may contribute to higher levels of early postoperative pain, potentially limiting early mobilization and delaying progression through the initial phases of rehabilitation. In contrast, preservation of tibial cortical integrity and reduced tissue disruption with the all-inside technique may facilitate a more comfortable early recovery period, allowing patients to advance through rehabilitation milestones more efficiently. Importantly, earlier return to sport in the all-inside group was not associated with a reduced rate of return to preinjury sport level, suggesting that accelerated recovery did not compromise functional readiness or safety.

Furthermore, knee stability outcomes support the equivalence of the two techniques. In the present study, postoperative Lachman and pivot-shift test results were comparable between groups at all follow-up intervals. These findings are consistent with biomechanical and clinical studies showing that suspensory fixation in all-inside ACL reconstruction provides stability comparable to traditional full tibial tunnel techniques [3,8,9,11,12]. The absence of differences in clinical stability indicates that reduced surgical invasiveness does not compromise biomechanical integrity.

A relevant clinical finding of this study is the consistently lower incidence of anterior knee pain in the all-inside group throughout follow-up. Anterior knee pain remains a common source of patient dissatisfaction after ACL reconstruction and has been associated with tibial tunnel-related morbidity and soft tissue irritation [5]. The decreased incidence observed in the all-inside group supports the tissue-preserving advantages of socket-based tunnel techniques and corroborates prior reports emphasizing improved patient comfort with minimally invasive approaches [5,10]. The consistently lower incidence of anterior knee pain observed in the all-inside group represents one of the most clinically meaningful findings of the present study. Anterior knee pain is a well-recognized cause of patient dissatisfaction after ACL reconstruction and may adversely affect daily activities, kneeling tolerance, and confidence during sport-specific movements. Preservation of the tibial cortex and avoidance of full-length tibial tunnel drilling with the all-inside technique may reduce irritation of periosteal and soft tissue structures around the proximal tibia, thereby mitigating anterior knee discomfort. Importantly, the sustained reduction in anterior knee pain observed at early, midterm, and final follow-up suggests that this benefit is not limited to the immediate postoperative period but may contribute to improved long-term patient comfort and satisfaction. From a clinical perspective, this finding supports the all-inside technique as a patient-centered approach, particularly for active individuals in whom anterior knee pain may delay rehabilitation progression or limit return to sport.

Revision surgery was required in a limited number of patients in both groups, with no significant difference between techniques. Although revision cases were excluded from the final functional outcome analysis, failure patterns were additionally considered using an intent-to-treat framework. Revision rates were low and comparable between groups, supporting the midterm durability of both techniques. Importantly, most revision procedures were performed following traumatic reinjury rather than technical insufficiency or graft-related failure. This observation suggests that revision risk in this cohort was more closely related to patient activity demands than to the reconstruction technique itself. Even though revision cases were excluded from the final analysis, the similarity in revision rates is in agreement with previously published midterm outcomes comparing all-inside and conventional ACL reconstruction techniques [7,13]. In the present cohort, graft-related failure due to insufficient graft diameter was unlikely, as all all-inside reconstructions were performed using quadrupled semitendinosus grafts with a minimum diameter of 8 mm. Potential bias related to non-randomized technique allocation and exclusion of revision cases from functional outcome analyses should be considered when interpreting these findings. While some systematic reviews and meta-analyses have revealed potential advantages of reconstruction over repair techniques regarding durability and failure rates, the current findings indicate that both reconstruction strategies provide acceptable midterm survivorship when appropriately applied [14].

Finally, ACL reconstruction outcomes may vary based on patient-specific factors, including sex and activity level. A recent systematic review emphasized sex-specific differences in clinical outcomes following ACL reconstruction, indicating that technique-related benefits may not be uniform across all patient subgroups [15]. This highlights the importance of individualized decision-making when selecting a surgical reconstruction technique. Based on these findings, the all-inside technique may be preferred for young, active patients in whom early pain control and timely return to sport are prioritized. The narrow age range and focus on young, physically active individuals may limit the external validity of these results to older or less active patient populations.

This study had several limitations. Its retrospective and the single center design and nonrandomized patient allocation introduce a potential risk of selection bias. The exclusion of patients with advanced concomitant meniscal or chondral lesions may limit the generalizability of the findings, particularly given the high prevalence of associated meniscal pathology among patients undergoing ACL reconstruction. Exclusion of revision cases from functional outcome analyses may have resulted in survivorship bias. Knee stability was assessed using clinical examination rather than instrumented laxity measurements. Differences in graft harvest techniques between groups, including preservation of the Gracilis tendon in the all-inside group, may have influenced early postoperative pain patterns and should be considered when interpreting pain-related outcomes. Additionally, although a standardized rehabilitation framework was applied, postoperative rehabilitation was individually tailored based on patient-specific recovery, which may have acted as a confounding factor influencing early clinical outcomes. Return to sport was based on clinical and functional criteria rather than biomechanical data. However, this homogeneous population may also be considered a strength, as it reduces variability related to age and activity level.

## 5. Conclusions

This study demonstrates that the all-inside ACL reconstruction technique offers clear early clinical advantages over the conventional full tibial tunnel approach. Patients treated with the all-inside technique exhibited significantly lower early postoperative pain, superior short-term functional outcomes, and a consistently lower incidence of anterior knee pain while achieving comparable knee stability and midterm clinical results. These benefits were observed without an increase in complications or revision rates. Our findings highlight the all-inside technique as a patient-centered surgical strategy that enhances early recovery, reduces anterior knee pain, and facilitates an earlier return to sport without compromising midterm clinical durability. Future prospective or better-adjusted studies are needed to confirm these findings and further clarify the long-term comparative effectiveness of all-inside and conventional ACL reconstruction techniques.

## Figures and Tables

**Table 1:** Demographic characteristics and follow-up duration of the Initial Study Cohort.

Parameters	Conventional Group (*n* = 58), *n* (%)	All-Inside Group (*n* = 46), *n* (%)	*p*-Value
**Age** (years) **Gender**	24.4 ± 3.5	23.1 ± 4.0	0.112 1.000
Male	52 (%89.7)	41 (%89.1)	
Female	6 (%10.3)	5 (%10.9)	
**Mean follow-up** (months)	52.4 ± 4.1	50.7 ± 3.7	0.056
**Trauma mechanism**, *n* (%)			0.930
Fall from height	8 (13.8%)	5 (10.9%)	
Sports injury	46 (79.3%)	39 (84.8%)	
Others	4 (6.9%)	2 (4.3%)	

Revision surgery 4 (6.9%) 4 (8.6%). Note: Patients requiring revision ACL surgery during follow-up were excluded from the final analysis. Revision surgery rates were similar between groups.

**Table 2:** Comparison of clinical outcome scores between the study groups.

	Conventional Group (Mean ± SD)	All-Inside Group (Mean ± SD)	*p*-Value
**Clinical evaluation**			
**VAS score**			
Pre-treatment	5.07 ± 0.95	4.88 ± 1.06	0.330
3rd month	3.22 ± 0.92	1.38 ± 0.49	<0.001
12th month	1.76 ± 0.9	1.89 ± 0.9	0.530
48th month (final follow-up)	0.44 ± 0.5	0.57 ± 0.5	0.290
**Lysholm Score**			
Pre-treatment	62.2 ± 8.24	60.72 ± 6.16	0.340
3rd month	71.0 ± 16.35	84.1 ± 17.10	0.002
12th month	83.0 ± 15.45	84.2 ± 15.10	0.710
48th month (final follow-up)	84.1 ± 15.05	84.6 ± 15.15	0.880
**IKDC Score**			
Pre-treatment	30.01 ± 3.66	29.40 ± 2.37	0.360
3rd month	70.20 ± 14.0	79.4 ± 17.10	0.008
12th month	76.43 ± 13.3	78.95 ± 15.10	0.460
48th month (final follow-up)	77.61 ± 14.2	79.40 ± 16.70	0.610
**Time to return to work** (days)	80.59 ± 18.6	77.11 ± 19.5	0.380
**Time to return to sport** (months)	16.40 ± 5.85	13.25 ± 2.70	<0.001
**Time to return to** **Pre-injury sport level (%)**	45 (83.3%)	34 (81.0%)	0.780

VAS: Visual Analog Scale; IKDC: International Knee Documentation Committee. Continuous variables were compared using an independent-samples *t*-test. Values are presented as mean ± standard deviation.

**Table 3:** (**A**). Preoperative knee stability findings (Initial Study Cohort). (**B**). Postoperative stability tests and anterior knee pain (Final Study Cohort). Final analysis only (revision cases excluded).

**(A)**			
**Parameter**	**Conventional Group (*n* = 58), *n* (%)**	**All-Inside Group (*n* = 46), *n* (%)**	***p*-Value**
**Pivot-shift test** (positive)	31 (53.4%)	22 (47.8%)	0.918
**Lachman test** (overall distribution)			0.559
Grade 3B	51 (87.9%)	43 (93.5%)	
Grade 2B	7 (12.1%)	3 (6.5%)	
**(B)**			
**Parameter**	**Conventional Group (*n* = 54), *n* (%)**	**All-Inside Group (*n* = 42), *n* (%)**	***p*-Value**
**Pivot-shift test** (positive)			
3rd months	2 (3.7%)	0 (0%)	0.503
**Lachman test—3rd month** (overall distribution)			0.620
Grade 1A	52 (96.3%)	40 (95.2%)	
Grade 2A	1 (1.9%)	0 (0%)	
Grade 2B	1 (1.9%)	2 (4.8%)	
**Anterior knee pain** (present)			<0.001 (for all)
3rd month	34 (63.0%)	5 (11.9%)	
12th month	28 (51.9%)	2 (4.8%)	
48th month (Final follow-up)	22 (40.7%)	1 (2.4%)	

Footnote: ACL: anterior cruciate ligament; n: number of patients. Preoperative stability assessments were performed in the initial study cohort prior to exclusion of revision cases. Only patients included in the final analysis (n = 54 and n = 42) were evaluated. Lachman test grades were classified as 1A, 2A, and 2B according to standard clinical criteria.

## Data Availability

The data supporting the findings of this study are not publicly available due to ethical restrictions and the protection of patient privacy.
